# Mechano-electric heterogeneity of the myocardium as a paradigm of its function

**DOI:** 10.1016/j.pbiomolbio.2015.12.007

**Published:** 2016-01

**Authors:** Olga Solovyova, Leonid B. Katsnelson, Peter Kohl, Alexander V. Panfilov, Andrey K. Tsaturyan, Pavel B. Tsyvian

**Affiliations:** a Institute of Immunology and Physiology, Ural Branch of the Russian Academy of Sciences, Ekaterinburg, Russia; b Ural Federal University, Ekaterinburg, Russia; c Research Centre for Cardiovascular Medicine, University of Freiburg, Germany; d National Heart and Lung Institute, Imperial College of London, UK; e Department of Physics and Astronomy, Ghent University, Ghent, Belgium; f Institute of Mechanics of Moscow State University, Russia; g Ural State Medical University, Ekaterinburg, Russia

**Keywords:** Cardiomyocyte, Myocardial tissue, Heart, Gradients in the cellular electrical and mechanical properties, Myocardial heterogeneity, Muscle duplex, Slow force response, Cardiac modeling, Wet and dry experiment

## Abstract

Myocardial heterogeneity is well appreciated and widely documented, from sub-cellular to organ levels. This paper reviews significant achievements of the group, led by Professor Vladimir S. Markhasin, Russia, who was one of the pioneers in studying and interpreting the relevance of cardiac functional heterogeneity.

“… So we have to make guesses in order to give any utility at all to science. In order to avoid simply describing experiments that have been done, we have to propose laws beyond their observed range.”

(“Messenger Lectures”, Richard Phillips Feynman, 1967).

## 1 Introduction: the myocardial heterogeneity challenge

Myocardial heterogeneity reveals itself at different spatial scales, ranging from the molecular to the organ level. Cardiac anatomy and histo-architecture are extremely complex, allowing the heart to effectively function as a pump. The complex geometry of myocardial tissue is associated with heterogeneity in regional stress and strain distributions. Differences in coronary vasculature and energy demand give rise to heterogeneity in regional metabolic conditions. Locally prevailing cell orientation (generally called ‘fibre orientation’) and organization into mechanically reinforced layers (‘sheetlets’) underlie significant anisotropy in electrical and mechanical properties of the tissue. In addition, there is spatio-temporal heterogeneity in electrical activation of different cardiac tissue regions, and non-homogeneity in active and passive deformation of tissue within the four cardiac chambers.

In the 1920s, Carl Wiggers was the first to focus on regional features of left ventricular (LV) wall motion during the contractile cycle. He found that the iso*volumic* phase of ventricular contraction is not ‘iso*metric*’. In particular, he observed that just before the onset of ejection, apical myocardium contracts, stretching basal myocardium that is activated later. He called this the ‘entrant phase’ of contraction and suggested that it may increase mechanical efficiency (Wiggers, 1927). Current experimental techniques have reconfirmed and characterized in more detail, the heterogeneity in spatio-temporal patterns of mechanical activation in different layers and regions of the LV wall (Ashikaga et al., 2007, Bogaert and Rademakers, 2001, Sengupta et al., 2006).

A corner-stone paper, published by Аrnold and Phyllis Katz, summed up the key concept (and apparent inherent contradiction) of modern myocardial heterogeneity research, in highlighting the emergence of “Homogeneity out of Heterogeneity” (Katz and Katz, 1989). In explaining this, the authors used ancient Greek triremes, very fast (for that time) warships with three banks of oars where each oar had a location-specific length and shape, as an example for a system that benefits from regional structural and functional heterogeneity to produce efficient overall function. Following this analogy, the authors suggested that “as in the trireme, the functionally homogeneous contraction of the muscular walls of the heart depends on heterogeneity in structure.” They hypothesised that the well-known morphological, electrophysiological, and metabolic differences in the heart involve adaptive molecular changes in cardiomyocytes to adjust their function to environmental conditions. Special attention was paid to the diversity in expression of myosin isoforms as one of the main determinants of contractile activity in myocardial cells.

Indeed, it is now evident that cardiomyocytes from various parts of the LV wall display molecular and cellular properties suitable for the different mechanical conditions they are exposed to. In the adult heart, electrophysiological heterogeneities exist along the apico-basal, left-right, and transmural axes, see articles by V. Markhasin et al. (e.g. Solovyova et al., 2014) and others (Antzelevitch and Fish, 2001, Bollensdorff et al., 2011, Cazorla and Lacampagne, 2011, Poelzing and Rosenbaum, 2004, Stelzer et al., 2008). The origin of electrophysiological heterogeneities of the adult heart has been suggested to lie in early cardiac development (Boukens et al., 2009). Owing to differences in expression levels of several molecular mechanisms, cells from sub-epicardial layers show faster contraction dynamics, shorter action potential (AP) durations (APD), and swifter Ca^2+^ transients (CaT) than (physiologically earlier-activated) sub-endocardial cells. Data on gradients in the APD in the apex-base direction are still insufficient, and appear to differ between species (Boukens et al., 2009, Janse et al., 2012). The transmural gradient in APD, found in isolated myocytes, is present in isolated perfused ventricular wedge preparations. In concert with the activation sequence, this may explain the absence of significant dispersion of repolarisation in the intact heart (Boukens et al., 2015, Myles et al., 2010, Taggart et al., 2003).

Pursuing the analogy by Katz & Katz with the trireme, such a heterogeneous system does not allow elements (oars) to change depending on either the instant external (sea) conditions or internal interactions between each other. However, it is becoming apparent now that cardiac heterogeneity involves highly dynamic interactions between (sub-)cellular and tissue levels.

In spite of an increasingly large body of data on cardiac molecular and cellular heterogeneity, its functional role remains largely under-appreciated, in part because it is difficult to visualise and experimentally explore regional tissue interactions. Significant progress in understanding the role of myocardial interactions at the tissue level has become possible with the introduction of paired muscle preparations in the late 1960s by Tyberg et al. (1969), subsequently expanded by Oscar H.L. Bing and co-workers (Shimizu et al., 1996, Wiegner et al., 1978). Both teams studied mechanical consequences of interaction between in-series connected normal and ischemic muscles well ahead of what is now referred to as the myocardial heterogeneity challenge – a focus of research of Professor Vladimir S. Markhasin (see brief CV in the electronic supplement).

V.S. Markhasin was one of the pioneers establishing the myocardial heterogeneity concept. Working on isolated myocardial preparations from human failing heart in 1970s and 80s, he observed remarkable heterogeneity in the cellular electrical activity of cells from single preparations (see Section 1). These observations led him to initiate research on cardiac heterogeneity both in normal and pathologically disturbed myocardium. Independently of the developments by Tyberg and others, he had developed paired muscle research in his lab in Ekaterinburg, Russia, calling it the ‘duplex’ method (Markhasin, 1983). The duplex approach has since been developed by his group for three decades (see Section 2). As the duplex system is the most simple representation of interaction myocardial regions, it has allowed the unraveling of basic properties of heterogeneous myocardium, as they emerge from element interactions. Applications of the heterogeneity concept to clinical research (see Section 3) and directions for future work based on the ideas of V.S. Markhasin are outlined below (see Conclusions).

## 2 Section 1: cellular heterogeneity in human heart

During his PhD research in the 1960s, V.S. Markhasin started (together with his friend and co-worker Valery Ya. Izakov) his scientific career by studying the electrical and mechanical activity of isolated myocardial preparations from frog heart (Izakov, 1967, Markhasin, 1967). At that time, they were among the first researchers in Russia who were able to measure cellular AP in cardiac muscle preparations using microelectrode techniques. In 1970s and 80s, V.S. Markhasin worked in the group of Professor Miloslav S. Savichevsky at the cardiosurgery department of the Sverdlovsk regional hospital where he pioneered research into isolated myocardial preparations from human heart, obtained during cardiac surgery. They used segments of atrial auricle and, occasionally, of LV from patients with congenital or acquired heart disease. Recording the mechanical activity of explanted tissue, using custom-made micro-mechanographic equipment, they found that preparations from failing heart developed significantly lower peak tension than normal myocardium, and that the time-course of contraction and relaxation were significantly slower (Grigorian et al., 1983, Markhasin and Tsivjan, 1980, Markhasin et al., 1981). Cellular AP, simultaneously recorded from several (25–70) cells of each preparation using floating microelectrodes, allowed the authors to assess variability in AP characteristics in the tissue. They observed less negative resting potentials (RP) in preparations from failing heart (Markhasin et al., 1981, Tsyv'ian and Markhasin, 1981). RP levels varied between cells from the same preparation by about 20%, independent of the disease. Corresponding fluctuations were seen in AP amplitude and time-course of depolarization (see Figure 1 in the e-supplement). In addition, myocardium from failing heart contained spontaneously excited cells. Effects of pacing rate, stretch, and inotropic agents on the recorded electrical and mechanical parameters were also investigated. Cells with different AP configurations responded differently to inotropic interventions, suggesting the presence of a clinically-relevant additional layer of heterogeneity – here in the effect of interventions to treat the failing tissue. Results of the comprehensive analysis of cellular activity in failing heart were summarized by V.S. Markhasin in his Doctor of Sciences thesis (Markhasin, 1983) and in a subsequent monograph (Markhasin et al., 1994b). Based on this work, V.S. Markhasin suggested that cellular remodeling in failing heart recruits embryonic mechanisms of excitation-contraction coupling, resulting in slower and less energy-demanding activity, which allows myocardial survival in pathological conditions. He conceptualized the processes underlying development of heart failure as being of mal-adaptive nature (Markhasin, 1983). These fundamental concepts would require more space to be discussed, and are outside the scope of this short tribute.

## 3 Section 2: tools to study the role of mechanical heterogeneity in myocardial function

Based on the observed heterogeneity in AP shape and duration in failing myocardium, the presence of heterogeneity in Ca^2+^ loading in cells was suggested to link this to heterogeneity in cellular mechanical activity. Another considered source of heterogeneity was associated with the shift in phases of contractile activity of muscle cells. This shift might be due to differences in the velocity of myocyte contraction and relaxation. Alternatively, the shift could be a consequence of a time delay in excitation (and, hence, onset of contraction) which may increase with a decrease in conduction velocity, for example. Another form of mechanical heterogeneity might also originate from, or be modulated by, exogenous or endogenous cardio-trophic interventions. In the 1980s, V.S. Markhasin suggested a conceptual approach (later named “Muscle Duplex”) for studying effects of myocardial mechanical heterogeneity, considering two mechanically interacting muscle elements (either two cells or two muscle segments) and discussing possible consequences of their mechanical asynchrony (Markhasin, 1983). In 1988, the approach to study paired muscle interactions was implemented, the experimental technique was patented ((Markhasin et al., 1990a), see also e-supplement for figures), and first results were published (Bliakhman et al., 1988, Bliakhman et al., 1989). At that time, Drs. V. Markhasin and V. Izakov worked together again, building a strong research group known as ‘the Ural school of myocardial biophysics and biomechanics', which subsequently formed a new department at the Institute of Physiology of the Ural Branch of Russian Academy of Sciences. In 1990s, the muscle duplex model of cardiac heterogeneity, together with results obtained from its application was published in several articles and monographs (Markhasin et al., 1990b, Markhasin et al., 1994b, Markhasin et al., 1999, Rutkevich et al., 1997).

In order to unravel underlying mechanisms, V.S. Markhasin turned to mathematical modeling, a powerful tool for predicting and analyzing complex systems (Bishop et al., 2013, Defauw et al., 2013, Hunter et al., 2001, Trayanova, 2011, Quinn and Kohl, 2013). V.Ya. Izakov and V.S. Markhasin had started to develop mathematical models of myocardium since the 70s (Izakov et al., 1991, Markhasin and Mil'shtein, 1978, Tsaturian and Izakov, 1978), an effort continued by his school to the present day (Katsnelson et al., 2014, Vasilyeva et al., 2014). A cellular model of the electrical and mechanical activity of cardiomyocytes (homogeneous cardiac muscle segment), developed by the Ekaterinburg team of V.S. Markhasin in collaboration with the Oxford team of Denis Noble, is referred to as the Ekaterinburg-Oxford model (EO model) (Solovyova et al., 2003, Sulman et al., 2008). Based on this model, theoretical muscle duplexes were built to predict effects of mechanical interactions of two virtual muscles, and to dissect possibly underlying sub-cellular mechanisms (Markhasin et al., 2003). Pushing the envelope even further, the team of V.S. Makhasin then used the EO model as part of direct biological experiments, in a set-up called “hybrid duplex”, where one biological muscle element interacts bilaterally and in real time with a virtual muscle (Protsenko et al., 2005). A comprehensive review of the various muscle duplex studies, implemented by V.S. Markhasin's team, was published last year (Solovyova et al., 2014), so here we briefly summarize only the most significant findings.

Muscle duplexes were used in six configurations: biological duplex (two coupled biological muscles), virtual duplex (two coupled virtual muscles) and hybrid duplex (one biological muscle coupled to a virtual muscle); each of these combinations has been implemented using either a parallel or an in-series mechanical connection between elements. Innovative features of the experimental duplex setting include the bidirectional mechanical communication between elements in real time (through computer interface signaling), while sustaining each element in separate and independent live preservation systems. Another distinguishing feature of the set of methods is the ability to directly compare/validate model predictions, obtained in the framework of virtual duplexes, with the experimental data registered in biological or hybrid duplex settings.

Initially, the duplex approach was thought to be useful for the study of myocardial heterogeneity in pathological conditions only, such as to explore interactions between normal and ischemic muscle segments, between segments with different grades/types of injury, or between segments exposed to different pharmacological interventions. It emerged, however, that myocardial heterogeneity is a physiological attribute of cardiac function in general, and that the duplex approach can provide a unique tool for the study of heterogeneity effects in normal tissue. Thus, muscle duplexes allow one to mimic interactions along the apex-to-base or endo-to-epi direction of ventricular tissue by changing activation delays of elements connected in-series or in-parallel, respectively. The muscle duplex approach allowed the group of V.S. Markhasin to unravel a new category of electrical and mechanical effects, caused by the myocardial heterogeneity of normal heart tissue, and to reveal the key role of mechano-electric feedback responses in tuning local mechanical contractility to global mechanical demand (Solovyova et al., 2014).

In all duplex settings (biological, virtual, and hybrid), mechanical coupling of two cardiac muscles causes significant changes in their mechanical behavior. Moreover as shown in virtual muscles and in biological preparations where either electrical activity or Ca^2+^ kinetics were recorded, that mechanical interactions between segments causes modulation of cellular Ca^2+^ in the interacting muscles. The specific direction and dynamics of responses depend on the sequence of muscle activation, mimicking possible variations in the delay and direction of the excitation wave in the heart. Several universal features were uncovered (Solovyova et al., 2014):

- *The tuning effect*, whereby interaction of muscle segment of in-parallel duplexes leads to functional fine-tuning of individual element mechano-electrical function. This was derived from results showing convergence of individual force/length, force/velocity, and force/useful work curves from in-parallel coupled duplex elements, even if the characteristics of uncoupled muscles were substantially different. The tuning of duplex activity towards optimal functional homogeneity occurred if the activation delay between muscle elements mimicked physiological conditions (i.e. later activation of the faster contracting muscle). The opposite (divergence of force/length and force/velocity behavior) was observed if in-parallel duplexes were exposed to an inverted activation sequence. The *tuning effect* highlights the delicate relationship between cellular properties and activation sequence in normal myocardium.
- C*ontractility conservation:* another common feature of in-parallel duplexes is that the force/velocity behavior of the duplex as a whole is little affected by the sequence of muscle stimulation and the time delay value. This occurs as a result of the reciprocal changes (convergence or divergence) of individual characteristics of the mechanical activity in interacting muscle segments. This phenomenon reflects high stability and adaptive reserve of normal heart muscle.
- *The intra-myocardial slow force response (SFR*_*IM*_*)*^1^ is a general feature of a heterogeneous myocardial system which was found during studies on in-series muscle duplexes (Markhasin et al., 2012). It occurs due to slowly developing cycle-by-cycle modulation in the contractile state of interacting muscle segments during their dynamic mechanical deformations. The SFR_IM_ results from the mechano-dependent changes in the cellular Ca^2+^ balance, recruiting mechanisms of mechano-calcium and mechano-electric coupling in cardiomyocytes. The original predictions from virtual duplexes have been confirmed in biological experiments, where mechanical activity of interacting muscle preparations was registered along with AP or CaT dynamics. Corresponding slow changes in contractile behavior were found in all duplex settings from a range of species (rat, rabbit, guinea pig), suggesting that they are not species-specific, but a general feature of (and an equalizer within) the heterogeneous myocardium (Markhasin et al., 2012).

Applying the duplex approach to pathological settings, a new explanation for ectopic excitation was found: ectopic beats may result (as a slow response) from mechanical interaction between cardiomyocytes with normal and moderately increased Ca^2+^ loading (Katsnelson et al., 2011).

Overall, data from duplex experiments suggest more complex roles for myocardial heterogeneity than just to serve as a static set of parameters that aid effective cardiac function. Instead, there is significant dynamic adjustment of regional electrical and mechanical properties, that can optimise the overall heart function (or, in pathological settings, contribute to mal-adaptation and arrhythmogenesis). In addition, heterogeneity cannot be understood in static systems, as *activation timing* is an essential input to regional interactions, where both *sequence* and *delay* of activation matter.

## 4 Section 3: translation of experimental findings to the beating heart

Based on results obtained in the experimental and theoretical duplex models V.S. Markhasin stressed that the tight interaction of cellular heterogeneity and activation sequence is of great clinical importance. For instance, the limited efficiency of implanted ventricular pacemakers, reportedly affecting up to 50% of patients, could be explained in part by dis-coordination of spatio-temporal heterogeneity in the heart. Efficiency of cardiac resynchronization therapy should essentially target recovery of near-normal coordination (not synchronization!) of activation dynamics and underlying cellular heterogeneity, as seen in the normal heart (Kirn et al., 2008). As presaged by V.S. Markhasin, dynamic deformations and cardiac mechano-electrical coupling have recently been demonstrated in the intact heart (Jeyaraj et al., 2007). It is now apparent that cardiac remodeling may be caused both by changes in cellular properties of the myocardium, and by abnormalities in excitation and contractions. This may be further enhanced by mechano-dependent tissue remodeling, via modulation in gene expression and protein synthesis (Berridge, 2003, Bers, 2011).

The data obtained from basic science research clearly show that quantitative characteristics of cardiac mechanical heterogeneity on the cellular, tissue and organ levels matter for the clinic. A separate direction of the research, pioneered by V.S. Markhasin, was focused on such translational efforts, putting the myocardial heterogeneity concept into clinical practice. In his work in the 1990s, conducted in collaboration with the Federal Research Center of Transplantology and Artificial Organs, he proposed an index of heterogeneity in LV regional movement, evaluated using conventional 2D echocardiography (Markhasin et al., 1994a). The coefficient of variation in the systolic reduction of LV sectoral areas was used as a measure of individual heterogeneity in LV tissue contraction. The heterogeneity index allows ones to distinguish between patients with cardiac disease from heart-healthy individuals. Later on, V.S. Markhasin introduced the term “functional geometry of the LV” to define coordination of dynamic changes in the geometry and mechanical activity of the LV during cardiac contraction (Chumarnaya et al., 2008). Several parameters of the segmental kinetics and dynamic change in the LV shape were evaluated in control individuals, in patients with ischemic heart disease with preserved global ejection fraction, and in patients with dilated cardiomyopathy (Chumarnaya et al., 2015, Chumarnaya et al., 2008). Significant distinctions were observed in functional geometry characteristics between the groups. This approach has since been applied to evaluating new-born babies (Ivanova et al., 2012), and studies are being continued by V.S. Markhasin's group in collaboration with the Ural State Medical University, the Sverdlovsk Regional Clinical Hospital, the Ural Research Institute of Mother and Child Care, Ekaterinburg, and the Bakoulev Scientific Center for Cardiovascular Surgery, Moscow.

Another source of heterogeneity in the human heart, addressed in V.S. Markhasin's studies, relates to heart rhythm variability and its effects on myocardial function. In his early work with musicians (for a period, he was employed as Professor in Physiology at the Ural State Conservatoire), he showed that pace and rhythm of musical phrases affected cardiac and respiratory frequency of listeners. “Quickening of phrase pulsation leads to increased excitement, while elongation of phrases calms, since almost every emotion has its characteristic type of breathing. Rhythmic organization of music governs also the rhythm of perception, modulating its intensity. Music affects the autonomic system in general, changing the heart rhythm.” (Markhasin and Tsehansky, 1978). These observations inspired study which for the first time demonstrated that physiological dispersion in pacing interval provides a positive inotropic effect on multicellular myocardial preparations compared to constant pacing (Izakov et al., 1983). V.S. Markhasin was one of the first to indicate that aperiodicity of cardiac excitation may be an important trigger of new adaptation mechanisms.

## 5 Conclusions and future research

Professor Vladimir Semionovich Markhasin contributed essentially to the concept and exploration of myocardial heterogeneity in normal physiology, in cardiac pathology, and in translation of basic science insight back to the clinic. His extensive development work on the muscle duplex approach, especially in combination with mechanical, electrophysiological and fluorescent measurement techniques, has given rise to an effective tool for the study of fundamental properties of heterogeneous myocardium. Closely integrated with computational modeling, it allows one not only to obtain and interpret new data, but also to make quantitative and experimentally testable predictions. More recently, the duplex approach has been extended by developing one-dimensional models of myocardial tissue, formed of electrically and mechanically coupled cardiomyocytes, which help to reveal effects of cellular interactions over larger spatial scales (Katsnelson et al., 2014). Also, his team has started to work on 3D models of LV electrical and mechanical heterogeneity, based on analytical descriptions of LV geometry and cell orientation (Pravdin et al., 2013). This is aimed at linking basic and clinical research even more closely, by adapting LV shape to personalized data obtained from echocardiography, CT, or MRI. The first steps in studying the role of LV geometry and myocardial anisotropy have been undertaken (Pravdin et al., 2015, Pravdin et al., 2014). This is a direction his team will continue to pursue within the new project “Personalized models in cardiology”, supported by the Russian Science Foundation (project 14-35-00005) – which had been initiated and led by the late V.S. Markhasin.

## Editors' note

Please see also related communications in this issue by Lang et al. (2016) and Opthof et al. (2016).

## Footnotes

1 We associated the response to muscle interactions with the ‘*slow force response*’ (Kentish and Wrzosek, 1998), which in our view represents a whole family of slow, stretch- (load)-induced changes in muscle contractility. This is different to, and distinct from, the immediate stretch-induced increase in contractility, like the Frank-Starling effect (Shiels and White, 2008), which occurs on the first beat following a change in preload, and in the absence of measurable changes in intracellular Ca^2+^ transients. The *intra-myocardial slow force response* is caused by dynamic interactions of muscle segments and, as the ‘typical stretch-induced SFR’, adjustment of the segments to altered mechanical conditions occurs over a number of beats (from tens to hundreds), until a new steady state is reached (Markhasin et al., 2012).
