# Supplementary material for: Mechano-electric heterogeneity of the myocardium as a paradigm of its function
Source: Prog Biophys Mol Biol. 2016 Jan;120(1-3):249–54. doi: 10.1016/j.pbiomolbio.2015.12.007 (PMC4821177; doi:10.1016/j.pbiomolbio.2015.12.007)
Supplement: Supplementary file 1 [file mmc1.doc]

**SUPPLEMENT**

for the article

“**MECHANO-ELECTRIC HETEROGENEITY OF MYOCARDIUM AS A PARADIGM OF ITS FUNCTION”**

by Solovyova O, Katsnelson L.B., Kohl P., Panfilov A.V., Tsaturyan A.K., Tsyvian P.B.

***In memory of Professor Vladimir Semionovich Markhasin***


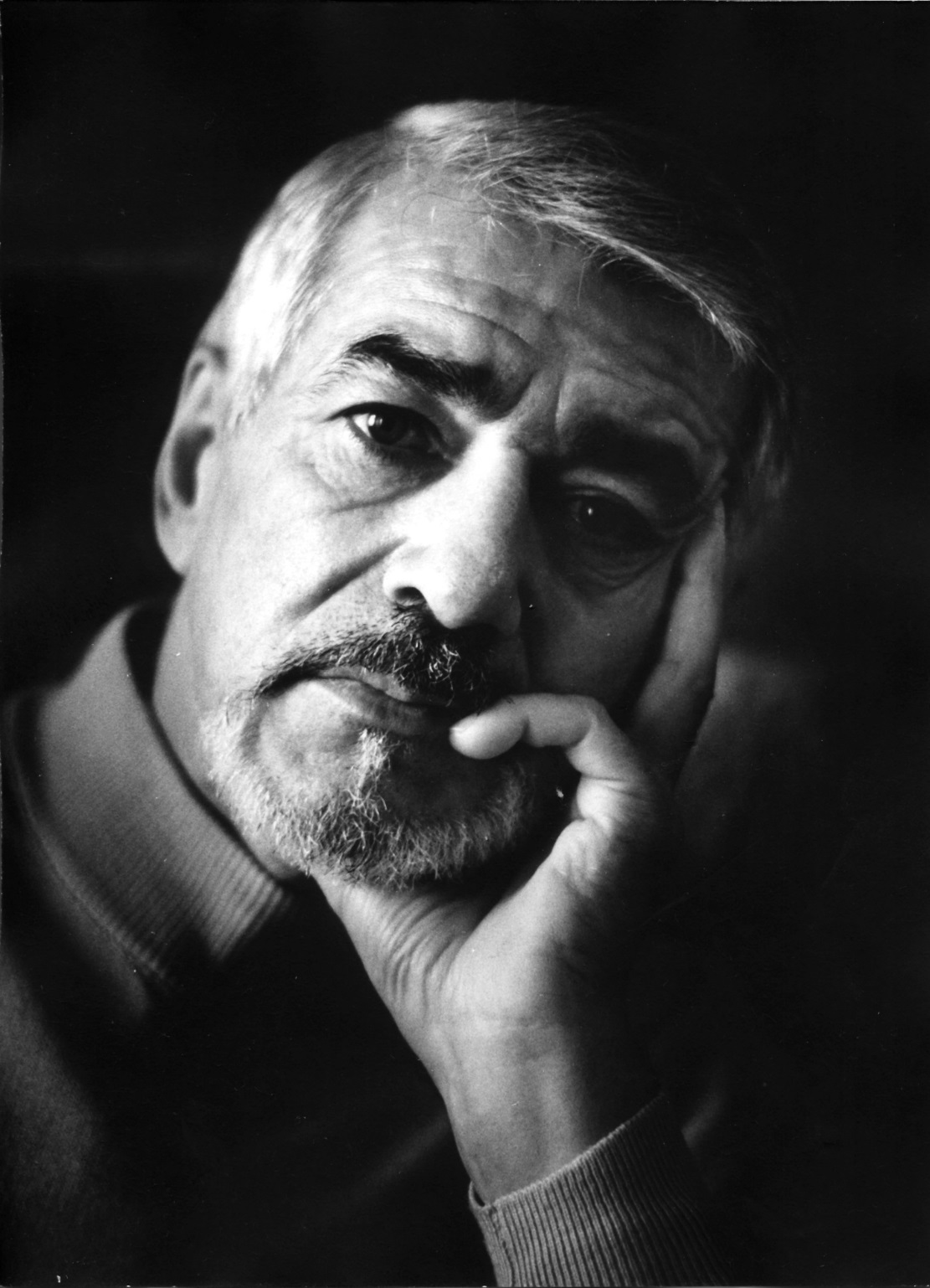


## Curriculum Vitae

**PERSONAL INFORMATION**

**Name & Titles Professor Vladimir Semionovich Markhasin**, MD, PhD, Dr. Sci., Corresponding Member of Russian Academy of Sciences

**Last Post** Principal Investigator at the Mathematical Physiology Laboratory at the Institute of Immunology and Physiology (IIF) of Ural Branch of Russian Academy of Sciences (UB RAS),

Professor in Biophysics at the Physics and Technology Institute of the Ural Federal University (UrFU)

**Dates of Life** 23 April 1941 - 11 April 2015

**Citizenship** Russian Federation

**ACADEMIC CAREER**

**2000 - 2015 Principal Investigator**Mathematical Physiology Laboratory, Institute of Immunology and Physiology UB RAS (Ekaterinburg, Russia)

**The Member of the Dissertation Council**Institute of Immunology and Physiology UB RAS (Ekaterinburg, Russia)

**Professor in Biophysics**Physics and Technology Institute, Ural Federal University (Ekaterinburg, Russia)

**1991 - 2000 Director**Ekaterinburg Filial of the Institute of Physiology RAS (Ekaterinburg, Russia)

**1988 - 1990 Head of Laboratory of Physiologically Active Substances**Institute of Physiology, UB of the USSR Academy of Sciences (Sverdlovsk, USSR)

**1979 - 1988 Senior Researcher**Biophysics Lab, Sverdlovsk Research Institute of Hygiene and Occupational Diseases (Sverdlovsk, USSR)

**1977 - 1979 Physician**Cardiac Surgery Department of the Sverdlovsk Regional Clinical Hospital (Sverdlovsk, USSR)

**1973 - 1977 Physician**Experimental Laboratory, Department of Cardiac Surgery, City Clinical Hospital #23 (Sverdlovsk, USSR)

**1967 - 1973 Assistant Professor**Department of Plant Physiology, Ural State University (Sverdlovsk, USSR)

**1964 - 1967 Junior Researcher**Central Research Physiological Laboratory, Sverdlovsk State Medical Institute (Sverdlovsk, USSR)

**1958 - 1964 Student**Sverdlovsk State Medical Institute (Sverdlovsk, USSR)

**DEGREES AND HONOURS**

**2012 Medal of the Order "For Merit to the Fatherland" II class** (Moscow, Russia)

**2003 Corresponding Member of Russian Academy of Sciences** in Physiology (Moscow, Russia)

**1999 Professor in Physiology,** Institute of Physiology UB RAS (Ekaterinburg, Russia)

**1995 Honored Scientist of the Russian Federation (Honorary Title in Russia)** (Moscow, Russia)

**1985** **Doctor of Sciences** in Biophysics (Moscow, USSR)

**1968**  **PhD** (Candidate of Sciences) in Physiology (Moscow, USSR)

**1964** **MD** (Dipl. med.), Sverdlovsk medical institute (Sverdlovsk, USSR)

**LAST RESEARCH FUNDING:**

**2014-2015** ***Personalized models in cardiology***
Russian Scince Foundation, #14-0005, PI: Markhasin V

**2012-2014** ***Three-dimensional computer model of the left ventricle to study the pathogenesis, diagnosis and prognosis of heart disease***
program for Basic Research of RAS Presidium of the strategic directions of development of science "Fundamental Problems of mathematical modeling", # 43П, PI: Markhasin V

**2012-2014** ***Molecular mechanisms of regulation of the kinetics of intracellular calcium in pacemaker cells and working myocardium in health and disease***
RAS Presidium program «The mechanisms of integration of molecular systems in implementing physiological functions», #12-П-4-1067, PI: Markhasin V

**2012-2014** ***The dynamics of the contractile function of the myocardium of ISIAH rat during the development of stress-induced arterial hypertension***
Program of supporting projects of collaboration between Ural, Siberian and Far Eastern Branches of Russian Academy of Sciencies, #12-C-4-1029, PI: Markhasin V

**2012-2014** ***Virtual Heart: Integrative Computer Mathematical Models***
Interdisciplinary project of UB RAS, # 12-М-14-2009, PI: Markhasin V

**2011-2013** ***Physiological and Pathophisiological Aspects of Pacemakers Implantation***
Project of oriented basic research of Ural Branch of RAS, #11-4-02-БИО, PI: Markhasin V

**2011-2013** ***Mechanisms of the contractile protein dysfunction in skeletal and cardiac muscle in hyperthyroidism***
Initiative RFBR grant, #11-04-00785-а, PI: Markhasin V

**2011** ***Organization of the Ural school of young scientists "Physiology and Biophysics of Myocardium", dedicated to the memory of Professor Izakov***
RFBR grant. Organization of scientific conferences and schools, #11-04-06804-моб_г, PI: Markhasin V

**2009-2011** ***The development of diagnostic indicators of regional heterogeneity of the kinetics of the wall of the left ventricle of the human heart during ontogenesis in normal and pathological conditions***
Programme of the Presidium of the RAS “Fundamental Science to Me”, #09-П-4-2010, PI: Markhasin V

**2008-2010** ***Role of the mechanical factors in disturbances of the electromechanical function of Ca2+ overloaded myocardium***
Initiative RFBR grant, #08-04-01137-а, PI: Markhasin V

**2006-2007** ***Molecular and cellular mechanisms of interaction of heterogeneous contractile elements of the myocardium***
Russian President Leading Scientific School Grant, #4923.2006.4, PI: Markhasin V

**2004-2009 *Mechanoelectrical Transduction in the Myocardium*** NIH-FIRCA, #1 R03_TW006250-01A1 PI: Markhasin V

**2004-2008 *Sub-cellular mechanisms of myocardial mechanical heterogeneity*** Wellcome Trust Collaborative Grant, #030588, PIs: Kohl P & Markhasin V

**2000-2003** ***Effects of mechanical inhomogeneity on myocardial contractile and electrical function***
Wellcome Trust Collaborative Grant, #061115, PIs: Kohl P& Markhasin V

**1997-2000** ***Active Myocardial Mechanics During Development and Diseases***
Civilian Research Development Foundation, #RN1-420, PI: Markhasin V

**PROFESSIONAL SOCIETIES**

**International** The Biophysical Society

**National** Russian Physiological Society

**REVIEWING AND EDITORIAL WORK**

**Editorial Boards** Russian Journal of Physiology, Russian Journal of Biomechanics, Journal Biomathematics and Bioinformatics

**Expert Panel** Russian Foundation for Basic Research , Russian Science Foundation


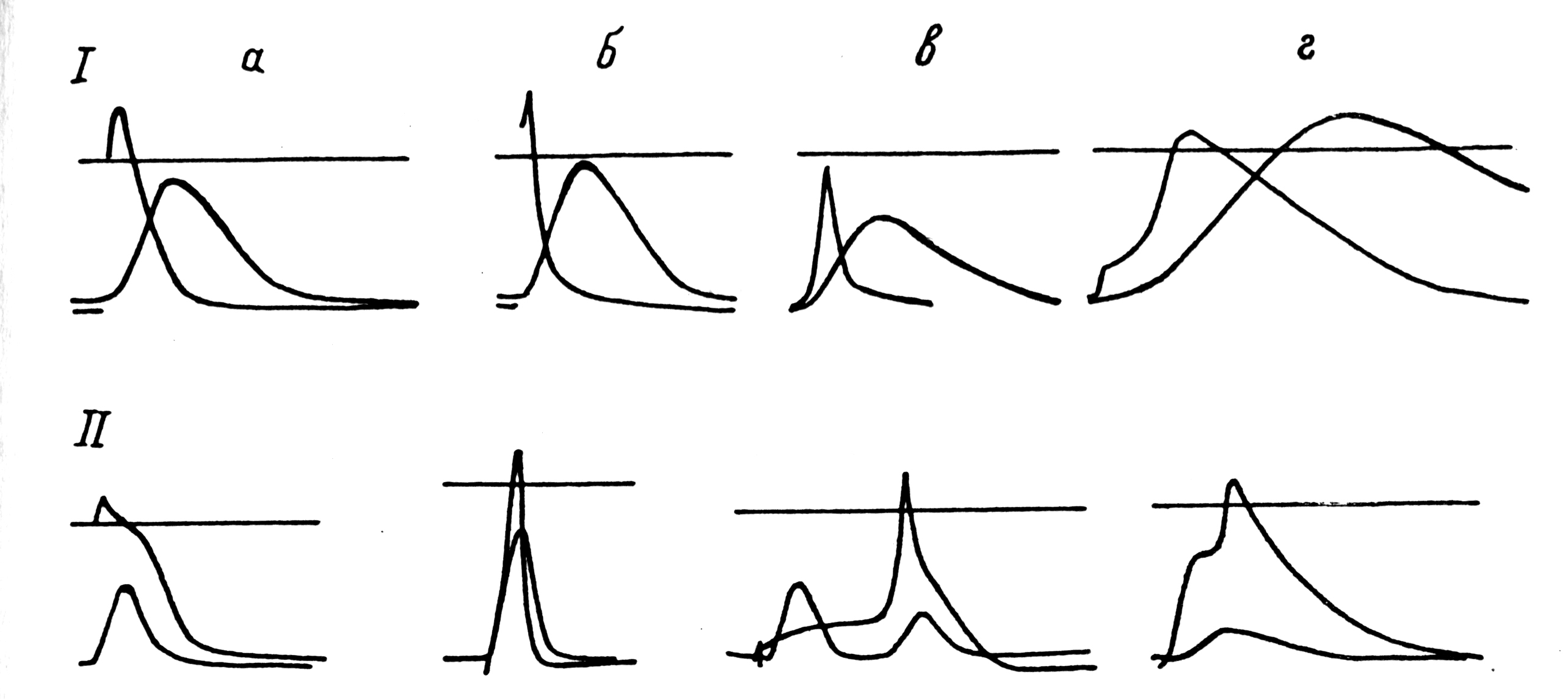


Figure 1. Representative recordings of the electrical and mechanical activity registered in preparations of the papillary muscle (top row) and atrial myocardium (bottom row) in patients with congenital and acquired heart diseases. From (Markhasin, 1983)


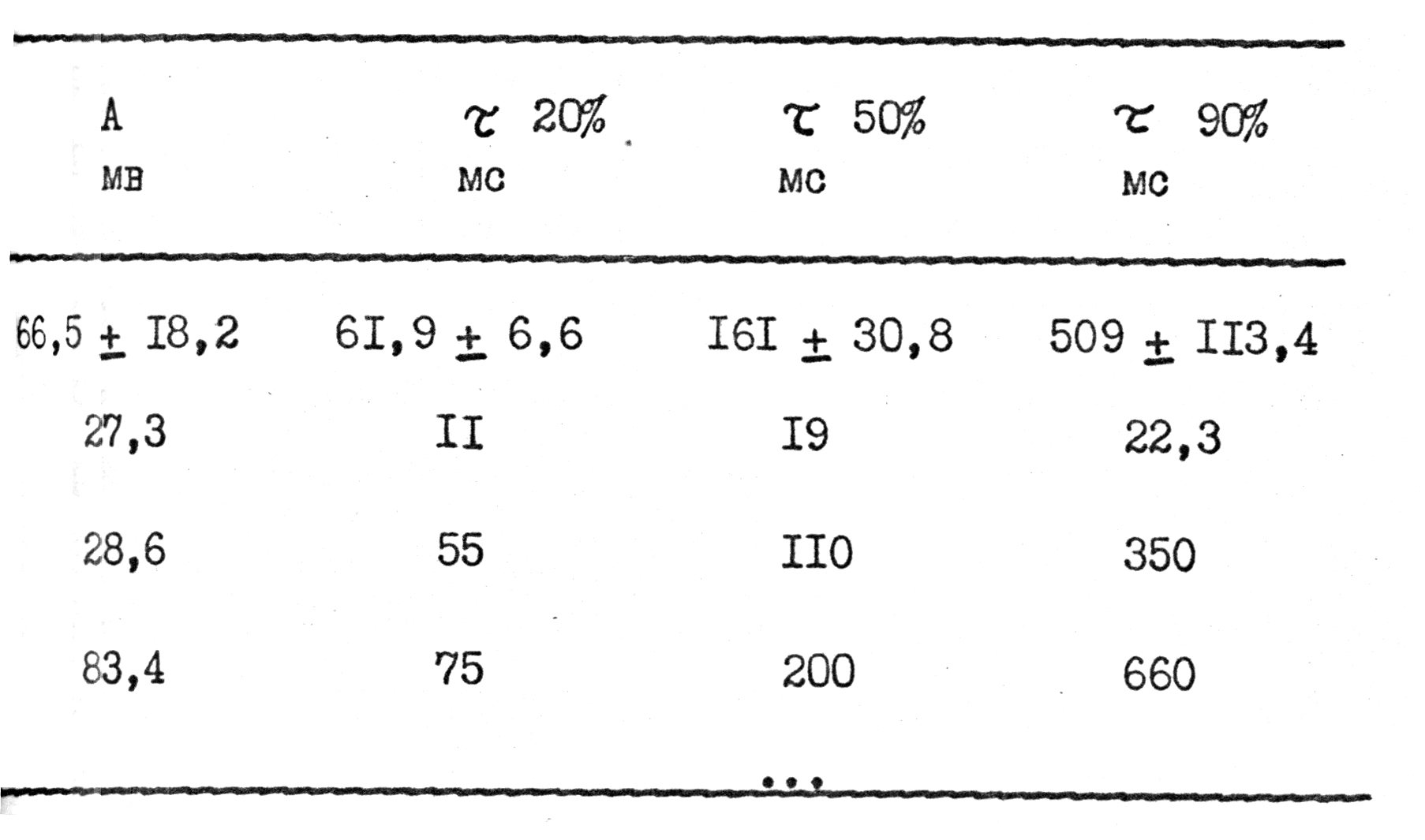


Figure 2. Statistical data on the parameters of AP registered in preparations from papillary muscles. A – AP amplitude (mV); τ – APD (ms). Top row: mean + standard deviation. Second row: coefficient of variation. Third row: minimum value. Bottom row: maximal value. From (Markhasin, 1983).


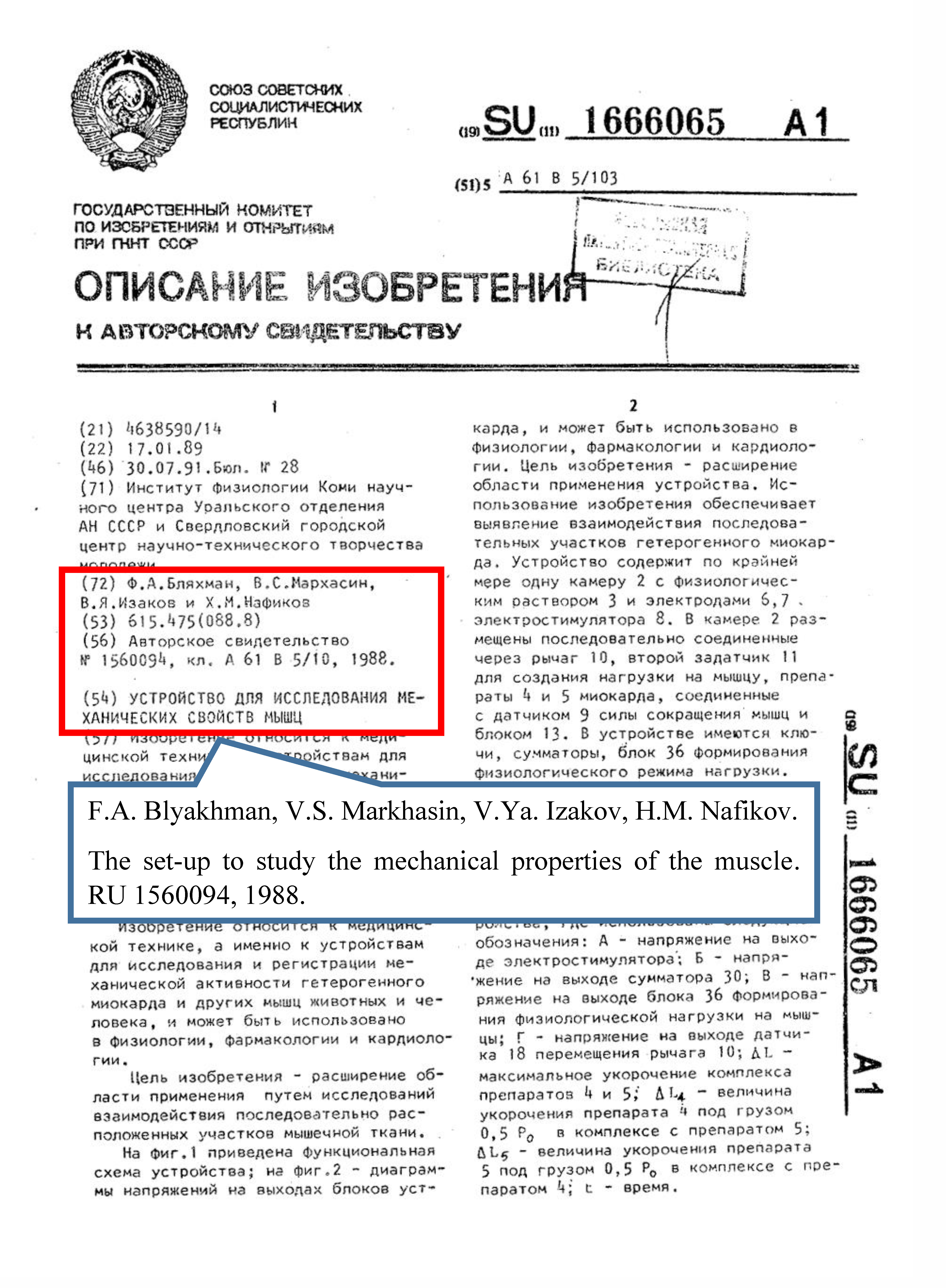


Figure 3. Patent for the set-up to study mechanical properties of muscles, 1988.


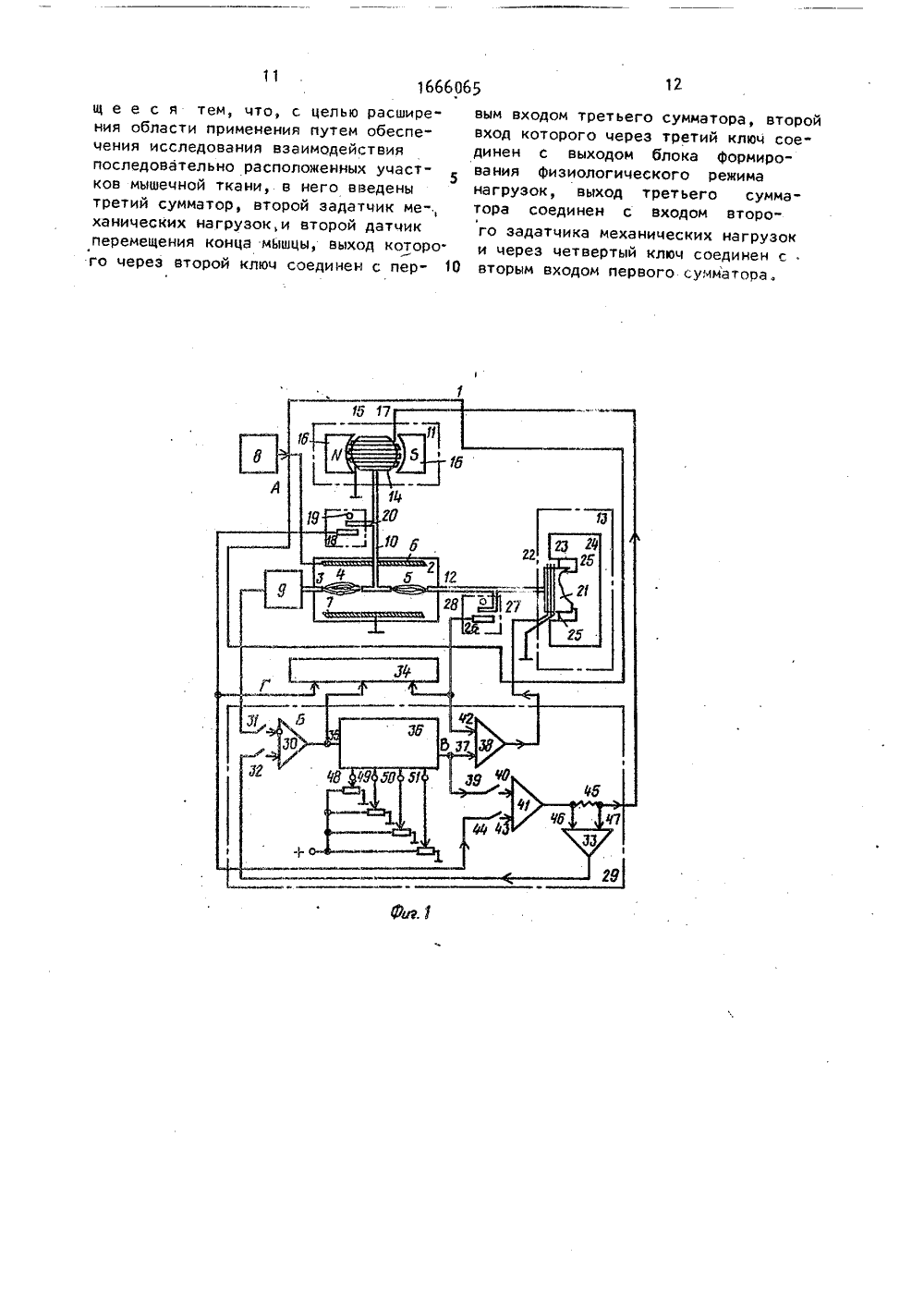


Figure 4. The scheme of muscle duplex set-up as it is shown in the patent technical description, 1988. It comprises at least one chamber (2) with physiological solution (3), electrodes (6, 7) of electric stimulator (8). The chamber 2 contains myocardial specimens (4,5), connected in-series through a lever (10), the second setting unit (11) for muscle deformations. The myocardial specimens (4) and (5) are connected with a force sensor (9) and the unit (13). The unit (13) has keys, summators, and a block to apply a physiological load.
